# Stanniocalcin‐1 Promotes PARP1‐Dependent Cell Death via JNK Activation in Colitis

**DOI:** 10.1002/advs.202304123

**Published:** 2023-12-13

**Authors:** Liguo Zhu, Zhuo Xie, Guang Yang, Gaoshi Zhou, Li Li, Shenghong Zhang

**Affiliations:** ^1^ Department of Gastroenterology The First Affiliated Hospital of Sun Yat‐sen University Guangzhou 510080 P. R. China; ^2^ Department of Minimally Invasive Intervention State Key Laboratory of Oncology in South China Guangdong Provincial Clinical Research Center for Cancer Sun Yat‐sen University Cancer Center Guangzhou 510060 P. R. China

**Keywords:** Colitis, JNK pathway, PARP1, parthanatos, STC1

## Abstract

Stanniocalcin‐1 (STC1) is upregulated by inflammation and modulates oxidative stress‐induced cell death. Herein, the function of STC1 in colitis and stress‐induced parthanatos, a newly identified type of programmed necrotic cell death dependent on the activation of poly‐ADP ribose polymerase‐1 (PARP1) is investigated. Results show that STC1 expression is markedly increased in the inflamed colonic mucosa of Crohn's disease (CD) patients and chemically‐induced mice colitis models. Evaluation of parthanatos severity and pro‐inflammatory cytokine expression shows that intestinal‐specific *Stc1* knockout (*Stc1* ^INT‐KO^) mice are resistant to dextran sulfate sodium (DSS)‐induced colitis and exhibit lower disease severity. STC1‐overexpressing cells show an increased degree of parthanatos and proinflammatory cytokine expression, whereas STC1‐knockout cells show a decreased degree of parthanatos. Co‐immunoprecipitation, mass spectrometry, and proteomic analyses indicate that STC1 interacts with PARP1, which activates the JNK pathway via PARP1–JNK interactions. Moreover, inhibition of PARP1 and JNK alleviates parthanatos and inflammatory injuries triggered by STC1 overexpression. Finally, following restoration of *Stc1* and *Parp1* expression by adeno‐associated viruses, and overexpression of *Stc1* and *Parp1* aggravated DSS‐induced colitis in *Stc1* ^INT‐KO^ mice. In conclusion, STC1 mediates oxidative stress‐associated parthanatos and aggravates inflammation via the STC1–PARP1–JNK interactions and subsequent JNK pathway activation in CD pathogenesis.

## Graphical abstract

A novel mechanism of colonic epithelial damage and parthanatos in patients with Crohn's disease (CD) is proposed. STC1 mediates oxidative stress‐associated parthanatos and aggravates inflammation via the STC1‐PARP1‐JNK interactions and subsequent JNK pathway activation in CD pathogenesis. The study provides a potential therapy for the alleviation of colitis.

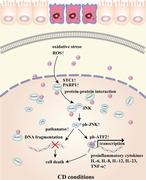

## 1 Introduction

As a chronic gastrointestinal inflammatory disease, Crohn's disease (CD) has complicated etiologies. It is well acknowledged that the interplay between genetics, environmental factors, and microbiota triggers an overactive mucosal immune response and gastrointestinal epithelial barrier damage.^[^ ^1^ ^]^ Under physiological conditions, the epithelial layer controls multiple functions, including nutrient recycling, mucus production, host‐microbial interactions, and mucosal immune response.^[^ ^2^, ^3^ ^]^ However, overactive immune and epithelial cells produce excessive reactive oxygen species (ROS) via pro‐inflammatory cytokine induction. Sustained ROS overload leads to a compromised intestinal barrier, DNA damage, and dysregulated cell death.^[^ ^4^ ^]^ Dysregulated necrotic cell death in intestinal epithelial cells further aggravates the pro‐inflammatory immune response and causes further damage to the epithelium.^[^ ^5^ ^]^

Stanniocalcin‐1 (STC1) is a glycoprotein that affects a broad spectrum of functions, including angiogenesis, tumorigenesis, and female reproduction.^[^ ^6^, ^7^, ^8^ ^]^ STC1 is significantly upregulated during inflammation and is stress‐responsive, increasing upon hydrogen peroxide (H_2_O_2_) stimulation.^[^ ^9^, ^10^, ^11^ ^]^ STC1 reportedly reduces oxidative stress by increasing mitochondrial uncoupling protein‐2 and inhibiting ROS‐induced apoptosis.^[^ ^12^, ^13^, ^14^ ^]^ Meanwhile, STC1 mediates cardiotoxicity by increasing ROS and inducing cell death in vitro and in vivo.^[^ ^15^ ^]^ Hence, existing literature indicates the potential role of STC1 as a stress responder and apoptosis mediator. Oxidative stress may induce multiple types of cell death, such as ferroptosis, cuproptosis, and parthanatos.^[^ ^16^, ^17^, ^18^ ^]^ However, the function of STC1 in these newly discovered types of cell death requires further investigation. In this study, we investigated the functioning mechanism by which STC1 mediates oxidative stress‐induced colonic epithelial damage and parthanatos in CD.

Among the multiple forms of cell death, parthanatos has received little research attention. Poly‐ADP ribose polymerase‐1 (PARP1)‐dependent cell death, or parthanatos, is initiated by PARP1 hyperactivation upon damage stimulation, such as oxidative stress and inflammatory stimuli.^[^ ^19^ ^]^ Poly (ADP‐ribose) (PAR) polymers are produced by overactivated PARP1 and are released from the nucleus into the cytosol. PAR binds to apoptosis‐inducing factor (AIF) and releases it from the mitochondria. AIF subsequently binds to macrophage migration inhibitory factor—a DNA nuclease—and translocates to the nucleus, leading to the cleavage of genomic DNA.^[^ ^20^ ^]^ As a type of programmed necrotic cell death, the occurrence of parthanatos is independent of the pro‐apoptotic caspase cascade and causes DNA breaks and cell membrane integrity loss.^[^ ^21^, ^22^ ^]^ Therefore, parthanatos exhibits a pro‐inflammatory feature in many diseases, including psoriasis and steatohepatitis.^[^ ^23^, ^24^ ^]^ Additionally, *Parp1* knockout mice exhibit defective nuclear factor‐κB (NF‐κB) activation and resistance to inflammation, including lipopolysaccharide‐induced septic shock and peroxynitrite‐induced arthritis.^[^ ^25^ ^]^ Furthermore, *Parp1* inhibition markedly alleviates colonic inflammation and protects the intestinal barrier in dextran sulfate sodium (DSS)‐induced and interleukin (IL)−10‐deficient spontaneous mouse models of colitis.^[^ ^26^, ^27^ ^]^

Based on existing evidence, the functions of STC1 and PARP1 in the CD pathogenesis remain unclear. Thus, in the current study, we investigate whether parthanatos contribute to the pathogenesis of oxidative damage in CD. Moreover, we elucidated the underlying mechanisms by which STC1 and PARP1 mediate oxidative stress‐induced colonic epithelial damage and parthanatos in CD.

## 2 Results

### 2.1 STC1 Expression is Upregulated in the Colonic Mucosa of CD Patients and Chemically‐Induced Murine Colitis Models

**Figure 1:**
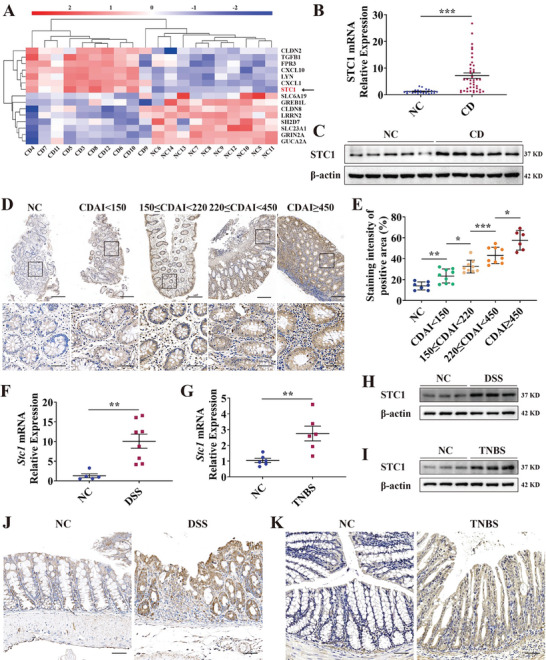
STC1 increases in the colonic mucosa of Crohn's disease (CD) patients and in chemically‐induced murine colitis. A) Heatmap of transcriptome sequencing showed STC1 mRNA increased in the inflamed colonic mucosa of CD patients (*n =* 10) compared with uninflamed colonic mucosa of healthy controls (*n =* 10). B) The expression level of STC1 mRNA in the inflamed colonic mucosa of CD patients (CD, *n =* 41) and uninflamed mucosa of healthy controls (NC, *n =* 25) was examined via qPCR. C) The expression level of STC1 protein in CD and NC colonic mucosa was detected by western blot. D) Representative IHC images of STC1‐stained colon sections from NC and different stages of CD. Scale bars: 200 µm (upper row) and 50 µm (lower row). E) Statistical analysis on the staining area of STC1 in NC and different stages of CD. NC, *n =* 7; CDAI<150, *n =* 9; 150≤CDAI<220, *n =* 9; 220≤CDAI<450, *n =* 9; CDAI≥450, *n =* 6. Positive staining area was quantified by ImageJ. F,G) The expression level of *Stc1* mRNA in DSS‐ (NC, *n =* 5; DSS, *n =* 8) and TNBS‐induced (NC, *n =* 6; TNBS, *n =* 6) mice colitis models were examined via qPCR. H,I) The expression level of STC1 protein in DSS‐ and TNBS‐induced colitis models was examined via western blot. J,K) Representative IHC images of STC1‐stained colon sections from DSS‐ and TNBS‐induced colitis models. Scale bars: 50 µm. Data were expressed as mean ± SEM. * *p* <0.05, ** *p* <0.01, *** *p* <0.001.

To identify the molecular factors in CD, we performed transcriptome sequencing of the inflamed mucosa from ten CD patients and normal mucosa from ten healthy controls (GSE230113), and a higher level of *STC1* mRNA expression was detected in the colonic mucosa of CD patients (**Figure** **1**A). These results were confirmed in an expanded sample size, and STC1 mRNA was markedly upregulated in inflamed CD and UC colonic mucosa compared with the uninflamed healthy mucosa (CD: 5.99‐fold, *p* < 0.001, Figure 1B, Table S3 (Supporting Information); UC: 4.20‐fold, *p* < 0.001, Figure S1A, Table S4, Supporting Information). Complementarily, the abundance of STC1 protein was markedly increased in the colonic tissues of CD patients (Figure 1C, Figure S1B, Supporting Information). STC1 expression also increased with increasing CD activity index (CDAI) (Figure 1D, E, Table S5, Supporting Information). Immunofluorescent staining revealed that STC1 was expressed in epithelial and immune cells. However, its expression is similar within the immune cells of inflamed and relatively normal colonic tissues of CD patients. In contrast, STC1 was upregulated in the epithelial cells from inflamed colonic tissues compared to the relatively normal CD colonic tissues. Accordingly, we focused on the function of STC1 in epithelial cells in subsequent analyses (Figure S1C, Supporting Information).

We also examined the expression of *Stc1* in both DSS‐ and TNBS‐induced acute colitis mouse models. *Stc1* mRNA was upregulated in DSS‐ (7.88‐fold, *p* < 0.01, Figure 1F) and TNBS‐induced murine colitis (2.64‐fold, *p* < 0.01, Figure 1G). Similarly, STC1 protein levels were increased in the colonic tissues of both colitis models (Figure 1H–K).

### 2.2 *Stc1* Deficiency Alleviates DSS‐Induced Acute Murine Colitis

**Figure 2:**
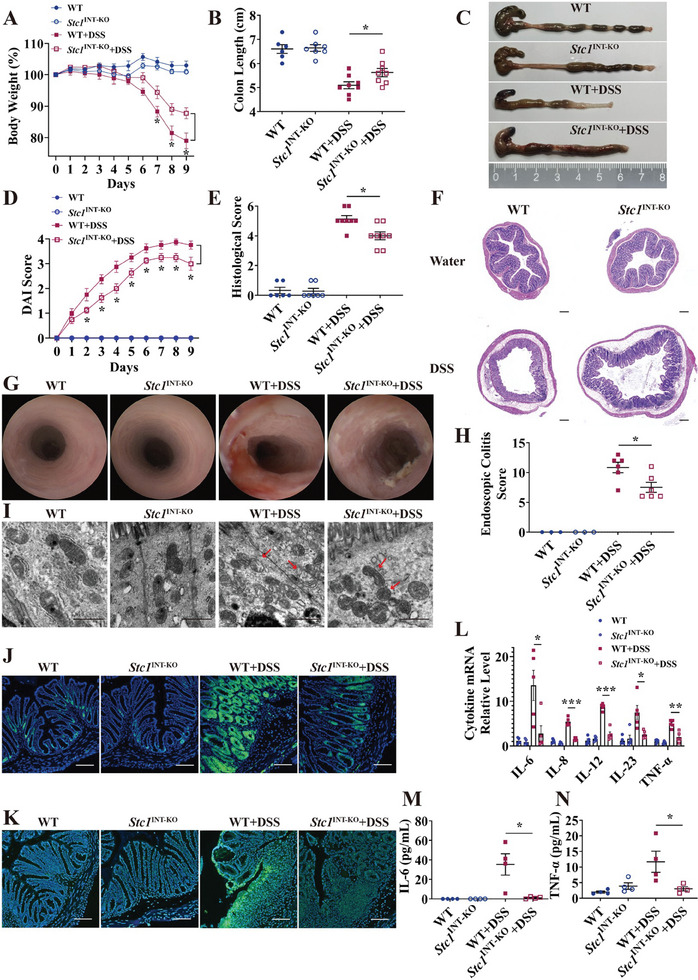
*Stc1* deficiency alleviates DSS‐induced acute murine colitis. A) Body weight loss of WT, *Stc1* ^INT‐KO^, WT+DSS, and *Stc1* ^INT‐KO^+DSS mice. WT, *n =* 6; *Stc1* ^INT‐KO^, *n =* 7; WT+DSS, *n =* 8; *Stc1* ^INT‐KO^+DSS, *n =* 8. (B, C) Mice colon length and representative images of colons. WT, *n =* 6; *Stc1* ^INT‐KO^, *n =* 7; WT+DSS, *n =* 8; *Stc1* ^INT‐KO^+DSS, *n =* 8. D) DAI scores were used in the evaluation of colitis severity. WT, *n =* 6; *Stc1* ^INT‐KO^, *n =* 7; WT+DSS, *n =* 8; *Stc1* ^INT‐KO^+DSS, *n =* 8. (E, F) Histological scores and representative colonic histopathological images. WT, *n =* 6; *Stc1* ^INT‐KO^, *n =* 7; WT+DSS, *n =* 8; *Stc1* ^INT‐KO^+DSS, *n =* 8. Scale bars: 200 µm. (G, H) Representative colonoscopy images and endoscopic colitis scores were used to evaluate colitis severity observed under murine colonoscopy. WT, *n =* 3; *Stc1* ^INT‐KO^, *n =* 3; WT+DSS, *n =* 6; *Stc1* ^INT‐KO^+DSS, *n =* 6. I) TEM detected the deformation of mitochondria (marked by red arrows) in mice colonic epithelial cells. Scale bars: 1 µm. (J, K) Representative IF images of γ‐H2AX‐ J) and PAR‐stained K) mice colon sections. Scale bars: 100 µm. (L) The expression level of pro‐inflammatory cytokine mRNA in mice colonic tissues was detected via qPCR. WT, *n =* 5; *Stc1* ^INT‐KO^, *n =* 5; WT+DSS, *n =* 5; *Stc1* ^INT‐KO^+DSS, *n =* 5. M,N) The expression level of IL‐6 and TNF‐α protein in mice serum was detected via multiELISA. WT, *n =* 4; *Stc1* ^INT‐KO^, *n =* 4; WT+DSS, *n =* 4; *Stc1* ^INT‐KO^+DSS, *n =* 4. Data were expressed as mean ± SEM. * *p* <0.05, ** *p* <0.01, *** *p* <0.001.

In DSS‐ and TNBS‐induced murine model of colitis, a substantial amount of ROS was produced (Figure S2A, Supporting Information). In this study, we used DSS to induce oxidative damage and inflammatory injury in mice colon. To investigate the function of *Stc1* in vivo, we generated intestinal‐specific *Stc1* knockout (*Stc1* ^flox/flox^: Villin Cre+, *Stc1* ^INT‐KO^) mice and littermate controls (*Stc1* ^flox/flox^, WT) (Figure S2B,C, Supporting Information). Compared with the WT+DSS mice, *Stc1* ^INT‐KO^+DSS mice exhibited less body weight loss, longer colon length, lower DAI scores, less histological damage in the colonic epithelium, and lower histological scores (**Figure** **2**A–F). Mild damage was observed in the colon wall of *Stc1* ^INT‐KO^+DSS mice under murine endoscopy, whereas WT+DSS mice exhibited marked changes in vascular patterns, increased mucosal granularity, and thickening of the bowel wall, which was quantified by the endoscopic colitis score (Figure 2G,H). Transmission electron microscopy (TEM) further revealed deformed and swollen mitochondria with fragmented cristae in the colonic epithelial cells of WT+DSS mice, whereas the mitochondria in the colonic epithelial cells of *Stc1* ^INT‐KO^+DSS mice were less damaged (Figure 2I). Additionally, phosphorylation of the H2A.X variant histone (γ‐H2AX) was used to show double‐stranded DNA breaks (DSBs). Lower levels of γ‐H2AX were produced in the colonic epithelium of *Stc1* ^INT‐KO^+DSS mice, indicating lower DSB frequency (Figure 2J, Figure S2D, Supporting Information). Meanwhile, PAR production was used to evaluate the degree of parthanatos, and it was markedly downregulated in *Stc1* ^INT‐KO^+DSS mice, suggesting that *Stc1* deficiency ameliorated parthanatos in DSS‐induced murine colitis (Figure 2K, Figure S2E, Supporting Information). Pro‐inflammatory cytokines, including IL‐6, IL‐8, IL‐12, IL‐23, and tumor necrosis factor (TNF)‐α, were significantly upregulated in the colonic tissues of WT+DSS mice, but this effect was weakened in *Stc1* ^INT‐KO^+DSS mice (Figure 2L). Serum IL‐6 and TNF‐α levels exhibited similar patterns in WT+DSS and *Stc1* ^INT‐KO^+DSS mice (Figure 2M,N).

### 2.3 STC1 Intensifies Parthanatos and Aggravates Inflammation In Vitro

**Figure 3:**
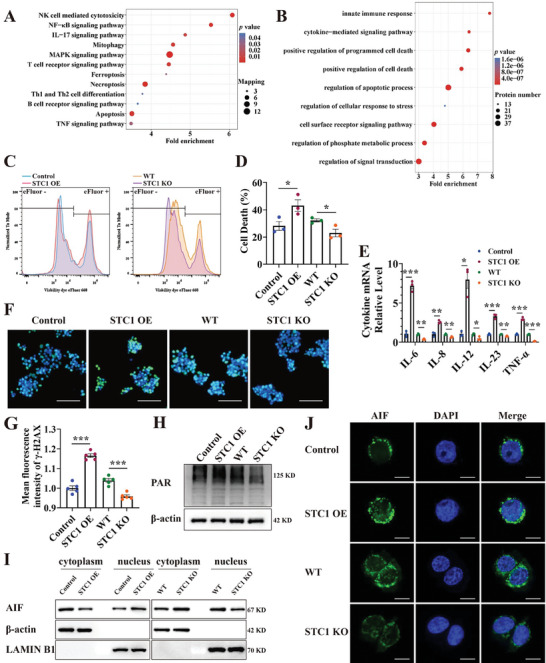
STC1 intensifies parthanatos and aggravates inflammation in vitro. A,B) KEGG A) and GO B) analysis on the enriched proteins in STC1 OE NCM460 cells. (C, D) Dye eFluor 660 marked dead cells after H_2_O_2_‐stimulation in Control, STC1 OE, WT, and STC1 KO NCM460 cells and was detected by flow cytometry. E) The expression level of pro‐inflammatory cytokine mRNA in H_2_O_2_‐treated NCM460 cells was detected via qPCR. F,G) Representative IF images and quantification of γ‐H2AX‐stained H_2_O_2_‐treated NCM460 cells. Scale bars: 100 µm. H) The expression level of PAR in H_2_O_2_‐treated NCM460 cells was detected via western blot. Scale bars: 100 µm. I,J) The nuclear translocation of AIF was detected via western blot I) and IF J). Scale bars: 10 µm. Data were expressed as mean ± SEM. * *p* <0.05, ** *p* <0.01, *** *p* <0.001.

In vitro, H_2_O_2_ was employed to trigger oxidative stress, and STC1 was upregulated upon H_2_O_2_ stimulation (Figure S2F,G, Supporting Information). To delineate the functional mechanisms of STC1 in vitro, we generated STC1‐overexpressing (STC1 OE) and STC1 knockout (STC1 KO) cell lines (Figure S2H–J, Supporting Information). Proteomic analysis of STC1 OE NCM460 cells revealed that STC1 upregulation was associated with pro‐inflammatory signaling, including NF‐κB, mitogen‐activated protein kinase (MAPK), TNF, and IL‐17 signaling pathways. Also, STC1 upregulation contributed to the enrichment of immune cell activation (e.g., T cell and B cell receptor signaling, and Th1/Th2 differentiation), and cell death (e.g., ferroptosis, necroptosis, and apoptosis) proteins in the Kyoto Encyclopedia of Genes and Genomes (KEGG) pathway analysis (**Figure** **3**A). Gene ontology (GO) analysis further revealed that biological processes, including the innate immune response, cell death, and stress response, were upregulated in STC1 OE cells (Figure 3B). Dye eFluor 660 was used to detect dead cells in H_2_O_2_‐treated Control, STC1 OE, WT, and STC1 KO NCM460 and Caco2 cell lines, revealing an increased proportion of dead cells in STC1 OE cells and a decreased proportion in STC1 KO cells (Figure 3C,D, Figure S3A,B, Supporting Information). Pro‐inflammatory cytokines, including IL‐6, IL‐8, IL‐12, IL‐23, and TNF‐α, increased in H_2_O_2_‐stimulated STC1 OE cells and decreased in STC1 KO cells (Figure 3E, Figure S3C, Supporting Information). Moreover, increased γ‐H2AX in STC1 OE NCM460 and Caco2 cells indicated that more DSBs occurred under oxidative stress, whereas less γ‐H2AX was observed in STC1 KO cells (Figure 3F,G, Figure S3D,E, Supporting Information). PAR synthesis was also upregulated in H_2_O_2_‐stimulated STC1 OE cells and inhibited in STC1 KO cells (Figure 3H, Figure S3F–H, Supporting Information). Similarly, AIF markedly translocated from the cytoplasm to the nucleus of STC1 OE cells, whereas this process was interrupted in STC1 KO cells (Figure 3I,J, Figure S3I,J, Supporting Information). We also assessed the well‐recognized markers of pyroptosis, ferroptosis, and necroptosis by western blotting in STC1 OE and STC1 KO cells. However, the expression of these markers was not significantly altered by STC1 overexpression or knockout in oxidative stress (Figure S3K, Supporting Information).

### 2.4 STC1 Interacts with and Upregulates PARP1 to activate the JNK pathway via PARP1–JNK Interaction in an Oxidative Stress‐Induced Inflammatory Environment

**Figure 4:**
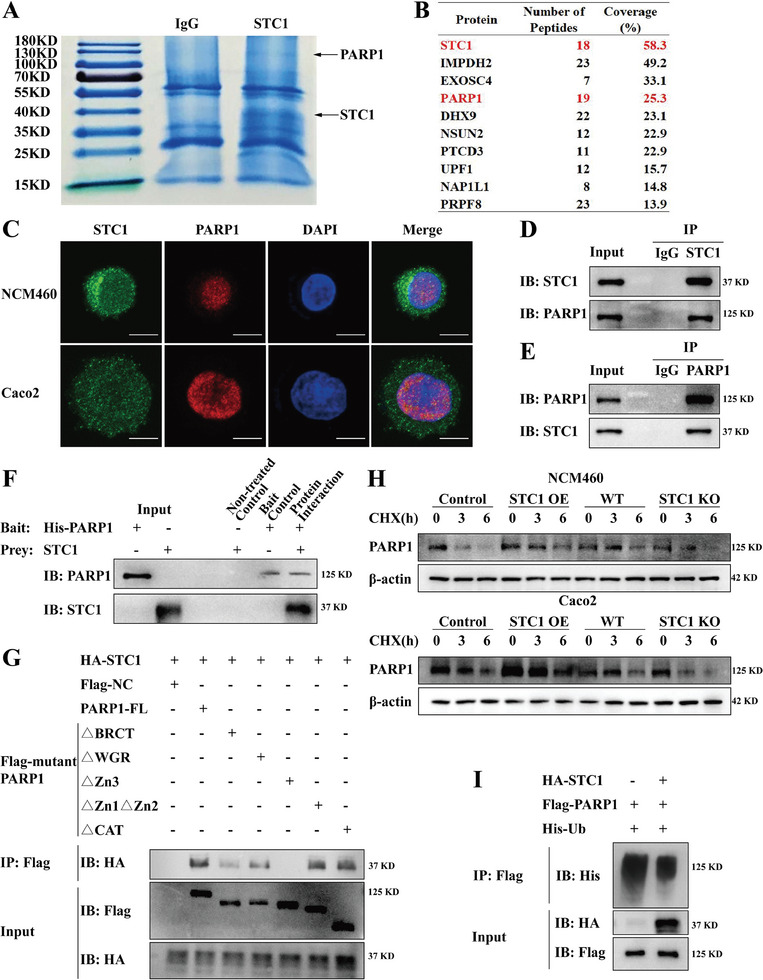
STC1 interacts with and upregulates PARP1. A) Coomassie blue was used in the preliminary identification of STC1 and target protein from the IP elution of STC1 OE NCM460 cells. B) Potential target proteins based on proteomic analysis. C) The subcellular distribution of STC1 and PARP1 protein in NCM460 and Caco2 cell lines was detected by confocal microscopy. Scale bars: 10 µm. (D, E) Anti‐STC1 D) and anti‐PARP1 E) antibodies were respectively applied in the Co‐IP of STC1 OE NCM460 cells. F) His pull‐down assays of purified recombinant human His‐PARP1 and STC1 protein. G) Protein truncated tests were designed to confirm the interaction between STC1 protein and PARP1 mutants in 293T cells. H) After H_2_O_2_‐pretreatment, the degradation of PARP1 protein in Control, STC1 OE, WT and STC1 KO NCM460 and Caco2 cells was detected via western blot, when protein synthesis was inhibited by 50µg mL^−1^ CHX in indicated time course. I) The ubiquitination of PARP1 protein was detected in the presence or absence of STC1 protein in 293T cells.

Immunoprecipitants from STC1 OE NCM460 cells were analyzed using high‐throughput mass spectrometry (MS) and proteomic analysis to identify proteins interacting with STC1. Therefore, we identified PARP1 as a potential candidate for interaction with the STC1 protein (**Figure** **4**A,B). We further analyzed the subcellular distribution of STC1 and PARP1 proteins and found that STC1 and PARP1 were co‐localized in the nucleus of NCM460 and Caco2 cells (Figure 4C, Figure S4A, Supporting Information). The STC1–PARP1 interaction was confirmed by co‐immunoprecipitation (co‐IP) and His pull‐down analysis (Figure 4D–F). Furthermore, we designed five PARP1 mutants. Protein truncation analyses revealed that full‐length PARP1 protein and truncated mutants lacking the first two zinc fingers (△Zn1△Zn2), the auto modification domain (△BRCT and △WGR), and the catalytic domain (△CAT) were able to bind STC1 protein. However, the △Zn3‐truncated mutant could not bind STC1, indicating that PARP1 probably interacted with STC1 via the Zn3 domain (Figure 4G, Figure S4B,C, Supporting Information).

In addition, we verified the upregulation of PARP1 protein in the colonic tissues of patients with CD (Figure S4D,E, Supporting Information) and in DSS‐ and TNBS‐induced acute mice colitis models (Figure S4F, Supporting Information). We then sought to determine the reason why PARP1 protein was upregulated by assessing whether STC1 affected PARP1 stability. Cycloheximide (CHX) was employed to inhibit protein synthesis. STC1 OE cells had higher PARP1 protein abundance after CHX treatment, whereas STC1 KO cells displayed rapid degradation of the PARP1 protein, indicating that STC1 caused PARP1 upregulation by maintaining PARP1 protein stability (Figure 4H). Furthermore, in the presence of STC1, PARP1 ubiquitination was decreased, suggesting that STC1 maintained PARP1 protein stability by inhibiting its ubiquitination (Figure 4I).

**Figure 5:**
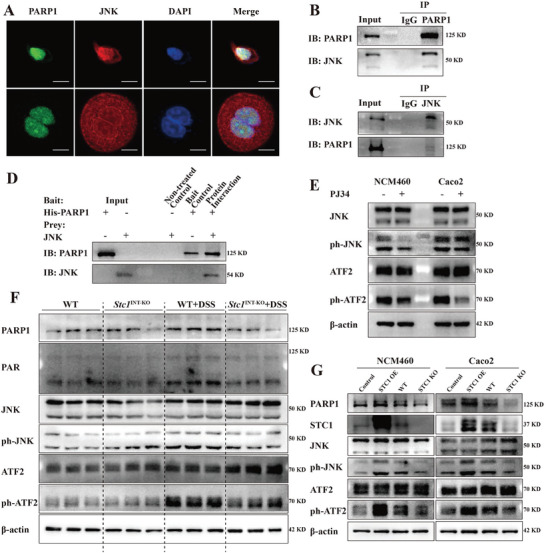
PARP1 activates JNK pathway via PARP1‐JNK interaction in an oxidate stress‐induced inflammatory environment. A) The subcellular distribution of PARP1 and JNK protein in NCM460 and Caco2 cell lines was detected by confocal microscopy. Scale bars: 10 µm. B,C) Anti‐PARP1 B) and anti‐JNK C) antibodies were respectively applied in the Co‐IP of STC1 OE NCM460 cells. D) His pull‐down assays of purified recombinant human His‐PARP1 and JNK protein. E) After the application of PARP inhibitor PJ34 (5µM, 24 h), the expression of JNK pathway protein in NCM460 and Caco2 cells was detected via western blot. F) The expression level of JNK pathway protein in the colonic tissues of WT, *Stc1* ^INT‐KO^, WT+DSS, and *Stc1* ^INT‐KO^+DSS mice was detected via western blot. G) The expression level of JNK pathway protein in H_2_O_2_‐treated STC1 OE and STC1 KO cells were detected via western blot.

Zhang S, et al reported that PARP1 binds to mitogen‐activated protein kinase 8 (JNK).^[^ ^28^ ^]^ Therefore, we examined the distribution of PARP1 and JNK proteins and observed their co‐localization in the nucleus of NCM460 and Caco2 cells (**Figure** **5**A). We also verified that PARP1 bound to JNK via co‐IP and His pull‐down analysis (Figure 5B–D). Considering that the JNK protein functions as a main effector in the JNK pathway, we further examined whether inhibition of PARP1 activity affected JNK pathway activation. After treating cells with the PARP inhibitor PJ34, phosphorylation of JNK and its downstream effector, ATF2, was markedly downregulated, suggesting that PARP1 inhibition impeded JNK pathway activation (Figure 5E). Given the enrichment of the MAPK signaling pathway in STC1 OE cells, we also assessed whether STC1 modulated the JNK pathway. The phosphorylation of JNK and ATF2 proteins was upregulated in WT+DSS mice compared with WT mice, but this effect was reversed in *Stc1* ^INT‐KO^+DSS mice (Figure 5F). Consistently, under H_2_O_2_ stimulation, PARP1, phosphorylated JNK, and ATF2 protein abundance were upregulated in STC1 OE NCM460 and Caco2 cells, whereas activation of the JNK pathway was inhibited in STC1 KO cells (Figure 5G).

These results indicate that STC1 induces PARP1 upregulation via the STC1–PARP1 interaction, and PARP1 further modulates JNK pathway activation via the PARP1–JNK interaction.

### 2.5 Inhibiting PARP1 and JNK Attenuates Parthanatos and Oxidative Stress‐Induced Inflammation

**Figure 6:**
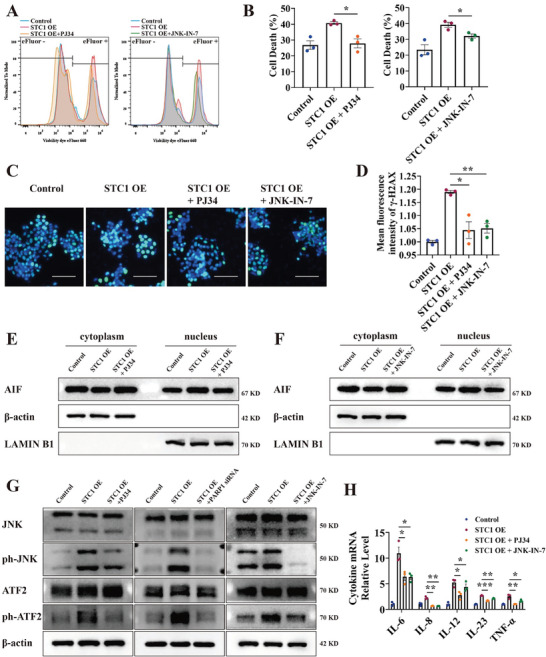
Inhibiting PARP1 and JNK attenuates parthanatos and oxidative stress‐induced inflammation. A,B) After pretreating cells with PARP inhibitor PJ34 (5µM, 24 h) or JNK inhibitor JNK‐IN‐7 (3µM, 24 h), eFluor 660 was used in the identification of dead NCM460 cells in oxidative stress and detected by flow cytometry. C,D) Representative images and quantification of γ‐H2AX‐stained H_2_O_2_‐treated NCM460 cells after PJ34 or JNK‐IN‐7 pretreatment. Scale bars: 100 µm. E,F) The nuclear translocation of AIF after PJ34 E) or JNK‐IN‐7 F) pretreatment was detected by western blot. G) The expression level of JNK pathway protein after treatment with PJ34, PARP siRNA, or JNK‐IN‐7 was detected by western blot. H) The expression level of pro‐inflammatory cytokine mRNA in H_2_O_2_‐treated NCM460 cells after PJ34 or JNK‐IN‐7 pretreatment was detected via qPCR. Data were expressed as mean ± SEM. * *p* <0.05, ** *p* <0.01, *** *p* <0.001.

We next investigated whether inhibiting PARP1 and JNK alleviates parthanatos and oxidative stress‐associated inflammation. After pretreating STC1 OE cells with the PARP inhibitor PJ34 and the JNK inhibitor JNK‐IN‐7, H_2_O_2_‐induced cell death was significantly reduced, accompanied by reduced DSB frequency (**Figure** **6**A–D, Figure S5A–D, Supporting Information). PJ34 and JNK‐IN‐7 decreased AIF nuclear translocation in STC1 OE NCM460 and Caco2 cells (Figure 6E,F, Figure S5E,F, Supporting Information). The application of PJ34, PARP1 siRNA, and JNK‐IN‐7 also inhibited JNK and ATF2 phosphorylation in vitro (Figure 6G, Figure S5G, Supporting Information). Furthermore, pro‐inflammatory cytokines, including IL‐6, IL‐8, IL‐12, IL‐23, and TNF‐α, were downregulated by PJ34 and JNK‐IN‐7 in H_2_O_2_‐stimulated STC1 OE cells (Figure 6H, Figure S5H, Supporting Information).

### 2.6 Restoring *Stc1* and *Parp1* Expression in *Stc1* ^INT‐KO^ Mice Aggravates DSS‐Induced Colitis

**Figure 7:**
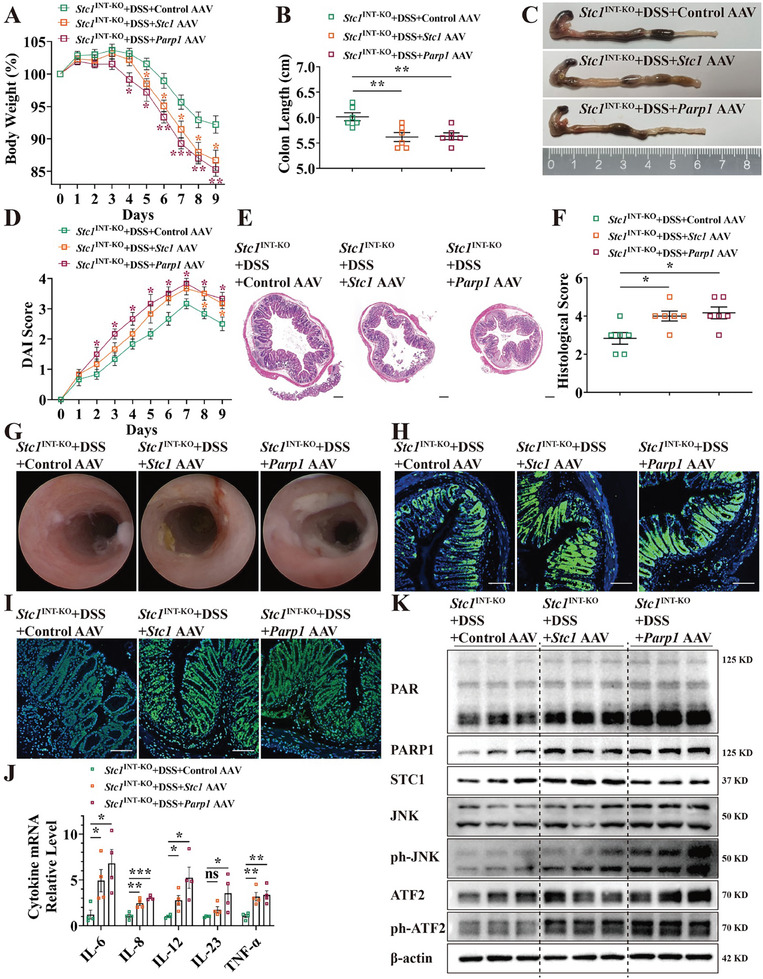
Restoring *Stc1* and *Parp1* in vivo aggravates DSS‐induced mice colitis. A) Body weight loss of DSS‐treated *Stc1* ^INT‐KO^ mice after peritoneal injection of *Stc1*‐overexpressing, *Parp1*‐overexpressing, or control AAV. *Stc1* ^INT‐KO^+DSS+Control AAV, *n =* 6; *Stc1* ^INT‐KO^+DSS+*Stc1* AAV, *n =* 6; *Stc1* ^INT‐KO^+DSS+*Parp1* AAV, *n =* 6. (B, C) Mice colon length and representative images of colons. *Stc1* ^INT‐KO^+DSS+Control AAV, *n =* 6; *Stc1* ^INT‐KO^+DSS+*Stc1* AAV, *n =* 6; *Stc1* ^INT‐KO^+DSS+*Parp1* AAV, *n =* 6. D) DAI score was used in the evaluation of colitis severity after AAV injection. *Stc1* ^INT‐KO^+DSS+Control AAV, *n =* 6; *Stc1* ^INT‐KO^+DSS+*Stc1* AAV, *n =* 6; *Stc1* ^INT‐KO^+DSS+*Parp1* AAV, *n =* 6. (E, F) Representative colonic histopathological images and histological scores. *Stc1* ^INT‐KO^+DSS+Control AAV, *n =* 6; *Stc1* ^INT‐KO^+DSS+*Stc1* AAV, *n =* 6; *Stc1* ^INT‐KO^+DSS+*Parp1* AAV, *n =* 6. Scale bars: 200 µm. G) Representative colonoscopy images were captured with murine colonoscopy. (H, I) Representative IF images of γ‐H2AX‐ H) and PAR‐stained I) mice colon sections. Scale bars: 100 µm. J) The expression level of pro‐inflammatory cytokine mRNA in mice colonic tissues was detected via qPCR. *Stc1* ^INT‐KO^+DSS+Control AAV, *n =* 4; *Stc1* ^INT‐KO^+DSS+*Stc1* AAV, *n =* 4; *Stc1* ^INT‐KO^+DSS+*Parp1* AAV, *n =* 4. K) The expression level of JNK pathway protein in mice colonic tissues was detected via western blot. Data were expressed as mean ± SEM. *p<0.05, **p<0.01, ***p<0.001.

We further investigated whether restoration of *Stc1* and *Parp1* aggravated murine colitis. To recover *Stc1* and *Parp1* expression, we intraperitoneally injected *Stc1* ^INT‐KO^ mice with AAV and induced colitis with DSS three weeks later. After restoring *Stc1* and *Parp1*, *Stc1* ^INT‐KO^ mice with DSS‐induced colitis exhibited increased weight loss, shorter colons, higher DAI scores, more histological damage in the colonic epithelium, and higher histological scores (**Figure** **7**A–F, Figure S6A,B, Supporting Information). Restoring *Stc1* and *Parp1* in vivo also led to more severe colonic mucosal damage during colonoscopy and higher endoscopic colitis scores (Figure 7G, Figure S6C, Supporting Information). Also, the indicators of parthanatos, including γ‐H2AX and PAR, increased in the colon of *Stc1* ^INT‐KO^ mice after *Stc1* and *Parp1* restoration (Figure 7H,I, Figure S6D,E, Supporting Information). TEM revealed markedly swollen mitochondria with fragmented cristae in colonic epithelial cells after *Stc1* and *Parp1* restoration (Figure S6F, Supporting Information). Additionally, the pro‐inflammatory cytokines IL‐6, IL‐8, IL‐12, and TNF‐α were increased in the colonic tissues of *Stc1*‐restored *Stc1* ^INT‐KO^ mice; these cytokines and IL‐23 were increased in the colonic tissues of *Parp1*‐restored *Stc1* ^INT‐KO^ mice (Figure 7J). However, significant changes were not observed in serum IL‐6 and TNF‐α levels. (Figure S6G,H, Supporting Information). The restoration of *Stc1* and *Parp1* also promoted PAR synthesis and JNK and ATF2 phosphorylation, indicating activation of the JNK signaling (Figure 7K).

## 3 Discussion

In our study, upregulation of STC1 intensified parthanatos and augmented pro‐inflammatory cytokine production in colonic epithelial‐like cell lines under oxidative stress, whereas STC1 deficiency reversed these phenotypes. *Stc1* ^INT‐KO^ mice showed resistance to DSS‐induced colitis and alleviated parthanatos. Furthermore, we verified that PARP1 acts as an interacting protein with STC1, and activates the JNK pathway by binding with JNK. Meanwhile, inhibiting PARP1 and JNK reversed the effects triggered by STC1 overexpression. Also, by restoring *Stc1* and *Parp1* expression, the observed resistance to DSS‐induced colitis in *Stc1* ^INT‐KO^ mice was abolished. Thus, STC1 participates in the pathogenesis of CD by binding to PARP1 and inducing parthanatos via JNK pathway activation.

Based on the existing evidence, STC1 has sophisticated functions in oxidative stress and inflammation. In oxidative stress, upregulated STC1 inhibits the pro‐survival effect of extracellular regulated protein kinase (ERK)−1/2 signaling and promotes fibroblast death.^[^ ^11^ ^]^ Also, STC1 aggravates cell apoptosis and autophagy by increasing JNK and p38 phosphorylation.^[^ ^29^, ^30^ ^]^ JNK, ERK, and p38 proteins belong to the MAPK family and participate in cell proliferation, differentiation, inflammation, and cell death.^[^ ^31^ ^]^ Therefore, STC1 may play an important role in MAPK signaling. Although STC1 has been defined as an apoptosis modulator, its function may be context‐dependent.^[^ ^32^, ^33^ ^]^ When cells are challenged with sublethal stress, STC1 reduces ATP and ROS production by uncoupling the mitochondria, while lethal stress excessively inhibits ATP production, and triggers cell death.^[^ ^34^ ^]^ Meanwhile, the role of STC1 in other types of cell death remains to be further investigated. Our work demonstrated that the upregulation of STC1 during inflammation may have a novel function in mediating parthanatos, therefore establishing a new role for STC1.

Via proteomic analysis, we observed enriched MAPK signaling in STC1‐overexpressing cells. As a member of the MAPK family, JNK signaling is activated by oxidative stress and inflammation, and its activation promotes cell aging and death.^[^ ^35^, ^36^ ^]^ JNK dysregulation leads to overactive immune responses and increased downstream pro‐inflammatory cytokine expression, correlating with immune disorders.^[^ ^37^ ^]^ Overactivation of the JNK pathway was confirmed in the inflamed colonic tissue of CD patients, and inhibition of JNK phosphorylation aids mucosal healing in CD.^[^ ^38^ ^]^ More specifically, activated JNK signaling enhances the translation and stability of pro‐inflammatory cytokine mRNAs, and mediates the production of IL‐6, IL‐8, IL‐12, IL‐23, and TNF‐α proteins, all of which have potential roles in CD.^[^ ^39^, ^40^ ^]^ Therefore, we selected the JNK pathway as the target and investigated its role in mediating parthanatos and inflammatory damage in colitis.

We found that the aforementioned pro‐inflammatory cytokines were downregulated in the colonic tissue and serum of *Stc1* ^INT‐KO^+DSS mice compared with WT+DSS mice. However, in the in vivo *Stc1* and *Parp1* restoration experiments, the serum cytokines tended to increase, but showed no significant statistical difference, whereas these cytokines significantly increased in colonic tissues. This may be due to the AAV peritoneal injection primarily affecting localized abdominal organs, with the applied dosage being insufficient to induce a significant general body response. However, this requires further investigation with dosage optimization.

Evidence also suggests that PARP1 binds to JNK protein.^[^ ^28^ ^]^ Post‐translational modification of PARP1(e.g., acetylation, ubiquitination, phosphorylation) can modulate its stability and activity.^[^ ^41^, ^42^ ^]^ In addition, PARP1 can impact its binding proteins by catalyzing PARylation. For example, PARP1 catalyzes the PARylation of the NF‐κB subunit p50 and p65, and activates NF‐κB for transcription.^[^ ^43^, ^44^ ^]^ We assumed that PARP1 activated JNK signaling via post‐translational modification of the JNK protein. However, the precise mechanism was not fully elucidated in our study, and warrants further investigation.

Recent evidence has shown that JNK activation promotes oxidative stress‐induced parthanatos.^[^ ^45^ ^]^ Therefore, we investigated whether JNK signaling mediates oxidative stress‐mediated parthanatos and inflammation in CD pathogenesis. Upregulated STC1 resulted in increased PARP1 and PAR, followed by parthanatos and JNK pathway activation in vitro and in vivo, which were subsequently rescued by inhibition of PARP1 and JNK pathways. Consistently, STC1 deficiency led to a corresponding inhibition of PARP1 activity, mitigated parthanatos damage, and reduced JNK pathway activation. In contrast, restoring *Stc1* and *Parp1* in vivo aggravated murine colitis. Mechanistically, we identified an STC1–PARP1–JNK interaction, which may be the basis of the observed effects.

Considering that PARP1 participates in DNA repair, PARP inhibitors have been developed to suppress DNA repair and induce cell death in cancer cells. Several PARP inhibitors, including olaparib, niraparib, rucaparib, and talazoparib, have been approved for clinical cancer treatment.^[^ ^46^ ^]^ Meanwhile, as PARP1 overactivation triggers parthanatos in lethal oxidative stress, PARP inhibitors may be cytoprotective. PARP inhibitors attenuate renal, cardiac, and intestinal ischemia‐reperfusion injuries.^[^ ^47^, ^48^, ^49^ ^]^ A new PARP inhibitor, JPI‐289, has entered clinical trials in ischemic stroke patients; preclinical data confirmed its efficacy in reducing infarct size and alleviating inflammation.^[^ ^50^ ^]^ However, the application of PARP inhibitors in inflammatory diseases is primarily limited to animal experiments. For example, olaparib alleviates pancreatitis and inflammatory cytokine production in murine models.^[^ ^51^ ^]^ Similarly, we showed that the application of the PARP inhibitor PJ34 alleviated oxidative stress‐induced parthanatos and inflammatory responses. These results were concordant with the anti‐inflammatory effect of PARP inhibitors in mouse models of colitis, suggesting that PARP inhibitors might be used in the treatment of inflammatory diseases.^[^ ^26^, ^27^ ^]^ Therefore, we anticipate the application of PARP inhibitors being applied in other non‐oncological diseases, particularly CD.

## 4 Conclusion

In summary, we demonstrated the important role of STC1 in mediating colonic epithelium parthanatos and oxidative stress‐associated inflammation via the STC1–PARP1–JNK axis in colitis (Figure S7, Supporting Information). STC1 may act as a player in the pathogenesis of colitis by inducing parthanatos. Hence, targeting STC1 and PARP1 might be a potential therapy for the alleviation of colitis.

## 5 Experimental Section

### Clinical Sample Collection

All human clinical samples were collected from the Department of Gastroenterology of the First Affiliated Hospital of Sun Yat‐sen University. Patients with CD were diagnosed according to the European Crohn's and Colitis Organization guidelines.^[^ ^52^ ^]^ Colonic biopsies of the healthy control group were collected during health checks and verified histopathologically.

### Conditional Knockout Mice and Chemically‐Induced Mouse Colitis

*Stc1*‐floxed C57BL/6J mice were generated using CRISPR/Cas9 techniques by the Shanghai Model Organisms Company, China. The mouse *Stc1* gene intron 1 was targeted by gRNA (5′‐GGGGTAGTGTCTTCTCCCTTAGG‐3′ and 5′‐CCAGAGAGAGCACGTGCCTAAGG‐3′). The mouse *Stc1* gene intron 2 was targeted by Grna (5′‐ AAGTTTAGACCAGGAATAAA GGG‐3′ and 5′‐ CAAGTTTAGACCAGGAATAA AGG‐3′).

*Stc1* ^flox/+^ mice were bred with Villin‐Cre mice to obtain *Stc1* ^flox/flox^: Cre+ and littermate control *Stc1* ^flox/flox^ mice. Mice were raised at 20–25 °C, 40–45% relative humidity, and a 12 h light/dark cycle in the specific pathogen‐free facility of the First Affiliated Hospital, Sun Yat‐sen University.

Mice (8–10 weeks old) were fed with 2.5% (w/v) DSS (MP Biomedicals, USA) solution for one week or were intracolonically administered with 2.5% (w/v) 2,4,6‐trinitrobenzenesulfonic acid (TNBS) (Sigma‐Aldrich, USA) to induce acute colitis, as described by Wirtz S, et al.^[^ ^53^ ^]^ Disease activity index (DAI) was evaluated based on the degree of weight loss, stool consistency, and intestinal bleeding. The histological scores were evaluated based on the degree of tissue damage and inflammatory cell infiltration. The endoscopic colitis score was evaluated based on colon thickening, vascular pattern, visible fibrin, mucosal surface granularity, and stool consistency.^[^ ^54^ ^]^ The adeno‐associated viruses (AAVs) used in the restoration of *Stc1* and *Parp1* were manufactured by ObiO Technology Corp., Ltd. (Shanghai, China). 2 × 10^11^ genomes of the AAV2/9 vector for *Stc1* and *Parp1* overexpression and control viruses were injected intraperitoneally into mice.

### Cell Culture

Caco2 cells were purchased from the American Tissue Culture Collection, USA, and cultured in Dulbecco's modified Eagle's medium supplemented with 10% fetal bovine serum (Gibco, USA). NCM460 cells were purchased from INCELL Corporation LLC, USA, and cultured in M3: BaseF medium (INCELL, USA) supplemented with 10% fetal bovine serum (Gibco, USA). Cells were cultured in a temperature‐controlled incubator at 37 °C and 5% CO_2_. Cells were treated with 500µM H_2_O_2_ for 12 or 24 h to induce oxidative stress.

### Antibodies and Primers

The antibodies used in this study are listed in Table S1 (Supporting Information). Primer sequences involved are listed in Table S2 (Supporting Information).

### Transmission Electron Microscopy

Fresh mice colonic tissues were harvested and made suitable for observation using TEM (HITACHI HT7800, Japan).

### Generation of STC1‐Overexpressing Cell Lines

The human STC1‐overexpressing lentivirus and control virus were synthesized by ObiO Technology Corp., Ltd. (Shanghai, China). After lentiviral transfection, cells were screened with 5 µg per mL puromycin for one week.

### Generation of the STC1 KO Cell Lines with CRISPR/Cas9

Complementary oligonucleotides for human STC1 gRNA (5′‐CACCGAGTCATTCTGCTCCGCCTCA‐3′ and 5′‐AAACTGAGGCGGAGCAGAATGACTC‐3′) were cloned into the GV392 CRISPR/Cas9‐Puro vector and used in lentiviral packaging by GENECHEM Corp., Ltd. (Shanghai, China). After transfection, cells were screened with 5 µg/mL puromycin for one week. Monocolonies were isolated and verified using DNA sequencing.

### PARP1 siRNA Transfection

PARP1 siRNA sequences: 5′‐GGCGAAGAAGAAAUCUAAATT‐3′; 5′‐UUUAGAUUUCUUCUUCGCCTT‐3′. PARP1 siRNA was transfected into cells with Lipofectamine RNAiMAX (#13778030) as per the manufacturer's instruction.

### Immunoprecipitation

A Pierce MS‐Compatible Magnetic IP Kit (#90409; Thermo Scientific, USA) was used for immunoprecipitation. Cell lysates were combined with IP antibody or control IgG and incubated at 4 °C overnight with gentle mixing to form an antibody‐antigen complex. Anti‐Flag Magnetic Beads (#HY‐K0207; MCE, USA) were used to immunoprecipitate Flag‐tagged protein. The immune complex was eluted from the beads and used for subsequent analysis.

### His Pull‐Down Assay

The Pierce His Protein Interaction Pull‐Down Kit (#21277; Thermo Scientific, USA) was used for His pull‐down assays. Purified recombinant human His‐tagged PARP1 protein (11040‐H08B; SinoBiological, China) was used as a bait protein. Recombinant human STC1 protein (#ab63280, Abcam, UK) and JNK protein (#TP322925, Origene, China) were prey proteins. Non‐treated gel control (minus bait, plus prey) and immobilized bait control (plus bait, minus prey) were used to eliminate false positives. The bait‐prey complex was eluted and used for western blotting.

### Mass Spectrometry Analysis

IP elution was digested and analyzed using tandem MS (MS/MS) in Q ExactiveTM Plus (Thermo Scientific, USA) coupled to the EASY‐Nlc 1000 UPLC system. The data were processed by the software Proteome Discoverer 1.3 (Thermo Scientific, USA).

### Nuclear and Cytoplasmic Extraction

Cytoplasm and nucleus proteins were separately extracted using Minute Cytoplasmic and Nuclear Extraction Kits for Cells (#SC‐003; Invent Biotechnologies).

### MultiELISA

Mice serum was collected for multiELISA analysis. Cytokines in mice serum were measured using the LXR‐MultiDTM‐10 kit, LabEx, China.

### Statistical Analysis

Statistical Product Service Solutions (IBM, USA) was used to perform statistical analyses. Two‐tailed Student's t‐test, one‐way analysis of variance and nonparametric Mann‐Whitney U test were used to assess differences between groups. Data were presented as mean ± standard error of the mean (n.s., no significance; *p<0.05; **p<0.01; ***p<0.001=. All data represented at least three independent experiments.

### Ethics Approval Statement

The collection of human clinical samples was approved by the Ethics Committee of the Institutional Board of the First Affiliated Hospital of Sun Yat‐sen University. Written informed consent was obtained from all of the participants. Animal experiments were approved by the Ethics Committee Institutional Board of the First Affiliated Hospital, Sun Yat‐sen University ([2021]747).

## Conflict of Interest

The authors declare no conflict of interest.

## Author Contributions

L.Z., Z.X., and G.Y. contributed equally to this work. S.Z., L.Z., Z.X., and G.Y. designed the study. L.Z., Z.X., and G.Y. performed the experiments. L.Z., Z.X., and G.Y. processed the experimental data and designed figures and tables in the manuscript. L.Z., Z.X., and G.Y. wrote the manuscript. S.Z., G.Z., and L.L. gave practical suggestions on the experiments and manuscript. All authors read and approved the final manuscript.

## Supporting information

Supporting Information

## Data Availability

The data that support the findings of this study are available on request from the corresponding author. The data are not publicly available due to privacy or ethical restrictions.
