# Supplementary material for: Stanniocalcin‐1 Promotes PARP1‐Dependent Cell Death via JNK Activation in Colitis
Source: Adv Sci (Weinh). 2023 Dec 13;11(5):2304123. doi: 10.1002/advs.202304123 (PMC10837357; doi:10.1002/advs.202304123)
Supplement: Supplementary file 1 — Supporting Information [file ADVS-11-2304123-s001.pdf]

ADVANCED SCIENCE
Open Access

Supporting Information

for *Adv. Sci.*, DOI 10.1002/advs.202304123

Stanniocalcin-1 Promotes PARP1-Dependent Cell Death via JNK Activation in Colitis

*Liguo Zhu, Zhuo Xie, Guang Yang, Gaoshi Zhou, Li Li and Shenghong Zhang**

Supplementary materials

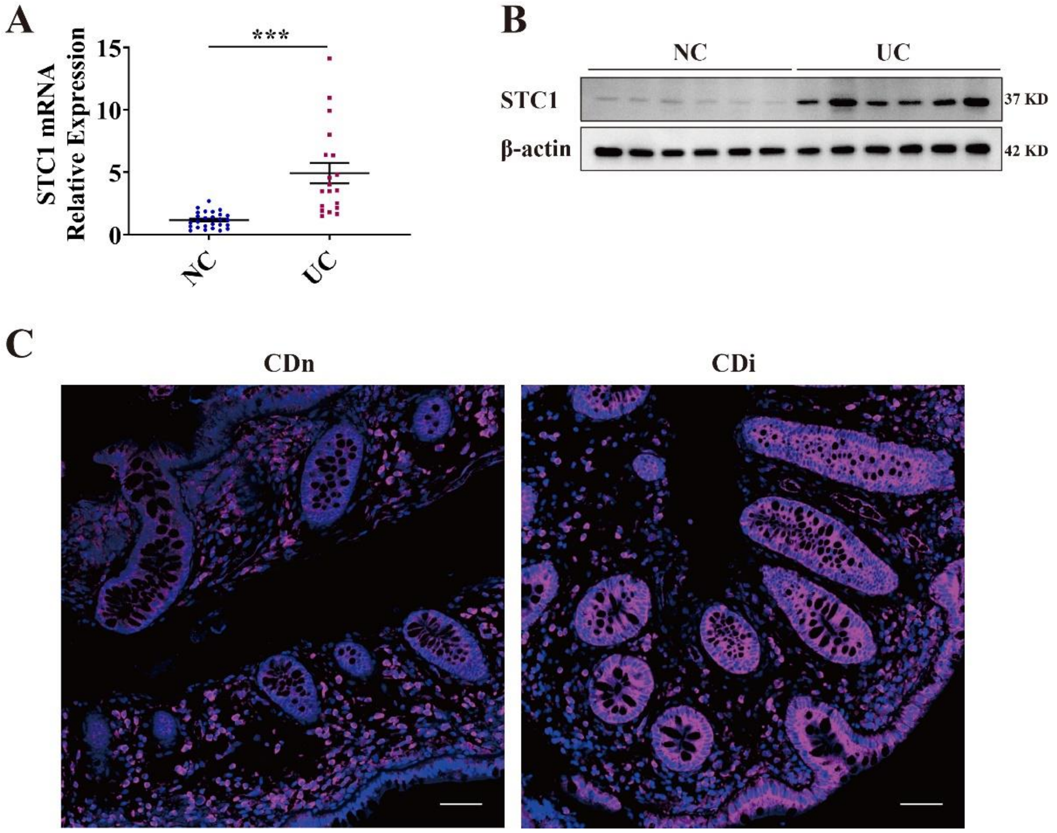

Figure S1. STC1 increases in the inflamed colonic mucosa of UC and CD patients

(A) The expression level of STC1 mRNA in the inflamed colonic mucosa of UC patients (UC, n=19) and uninflamed mucosa of healthy controls (NC, n=26) was examined via qPCR. (B) The expression level of STC1 protein in UC and NC colonic mucosa was detected by western blot. (C) Representative IF images of STC1-stained relatively normal and inflamed colonic tissues from CD patients. Scale bars: 50 μm. Data were expressed as mean ± SEM. * p <0.05, ** p <0.01, *** p <0.001.

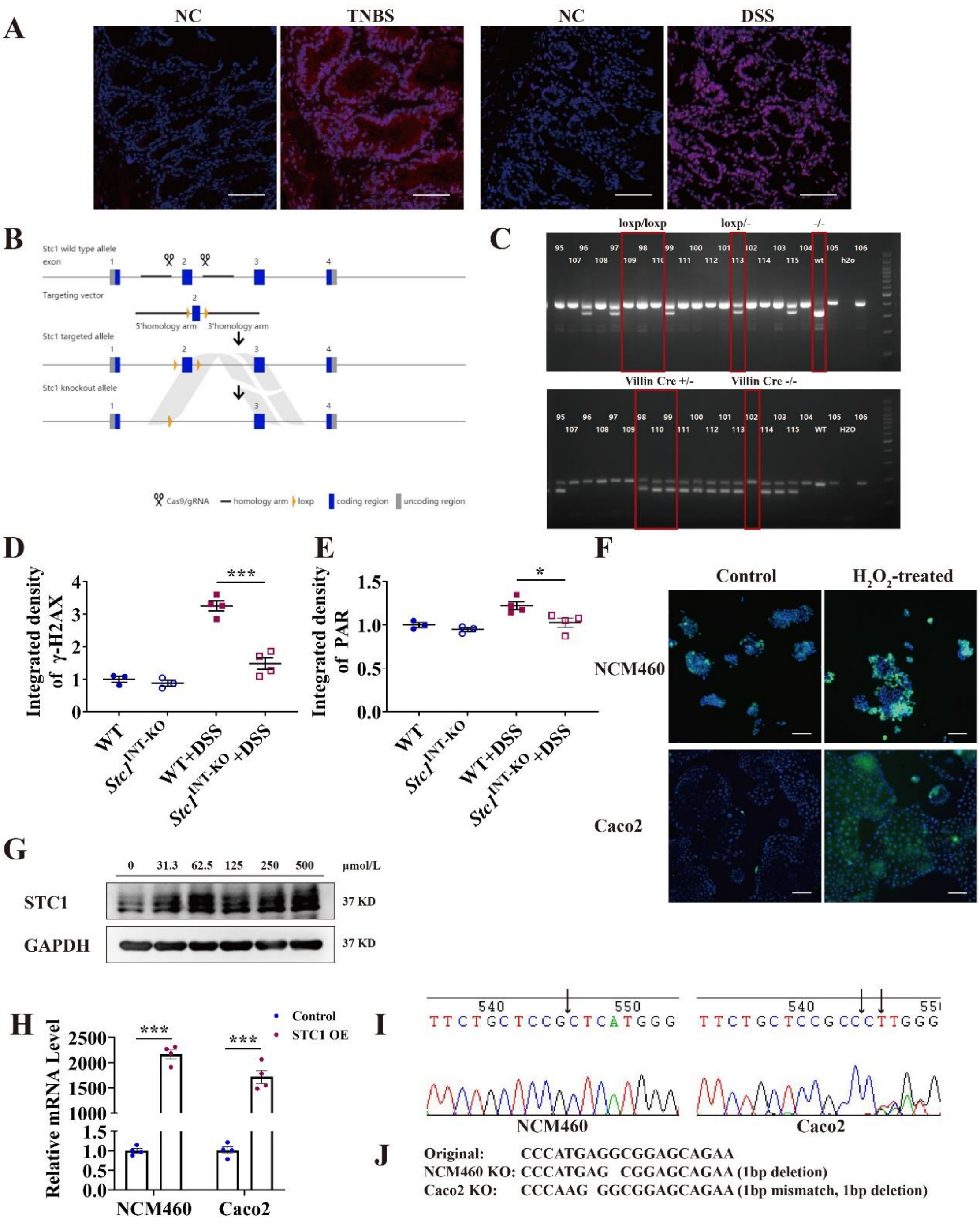

Figure S2. Genomic identification of *Stc1*$^{\text{INT-KO}}$ mice and STC1 KO clones.

(A) ROS production in the colonic tissue of TNBS- and DSS-induced colitis models was detected by ROS staining. Scale bars: 100 μm. (B) Schematic illustration of the generation of *Stc1*$^{\text{INT-KO}}$ mice. (C) Genomic identification of *Stc1*$^{\text{INT-KO}}$ (*Stc1*$^{\text{flox/flox}}$: Villin Cre+) and WT (*Stc1*$^{\text{flox/flox}}$) mice. (D) The quantified fluorescent density of γ-

H2AX-stained mice colon sections. WT, n=3; *Stc1*$^{\text{INT-KO}}$, n=3; WT+DSS, n=4; *Stc1*$^{\text{INT-KO}}$+DSS, n=4. (E) The quantified fluorescent density of PAR-stained mice colon sections. WT, n=3; *Stc1*$^{\text{INT-KO}}$, n=3; WT+DSS, n=4; *Stc1*$^{\text{INT-KO}}$+DSS, n=4. (F) ROS production in H_2O_2-treated NCM460 and Caco2 cells was detected by ROS staining. Scale bars: 100 μm. (G) The expression level of STC1 protein in H_2O_2-treated NCM460 cells was detected by western blot. (H) The expression level of STC1 mRNA in STC1 OE NCM460 and Caco2 cells. (I) DNA sequencing data of STC1 mutation in NCM460 and Caco2 clones. Black arrows indicated the heterogeneous genomic DNA sequences of the clones. (J) CRISPR-Cas9 introduced deletion and mismatch mutation in the targeted sites of NCM460 and Caco2 cells. Data were expressed as mean ± SEM. * p <0.05, ** p <0.01, *** p <0.001.

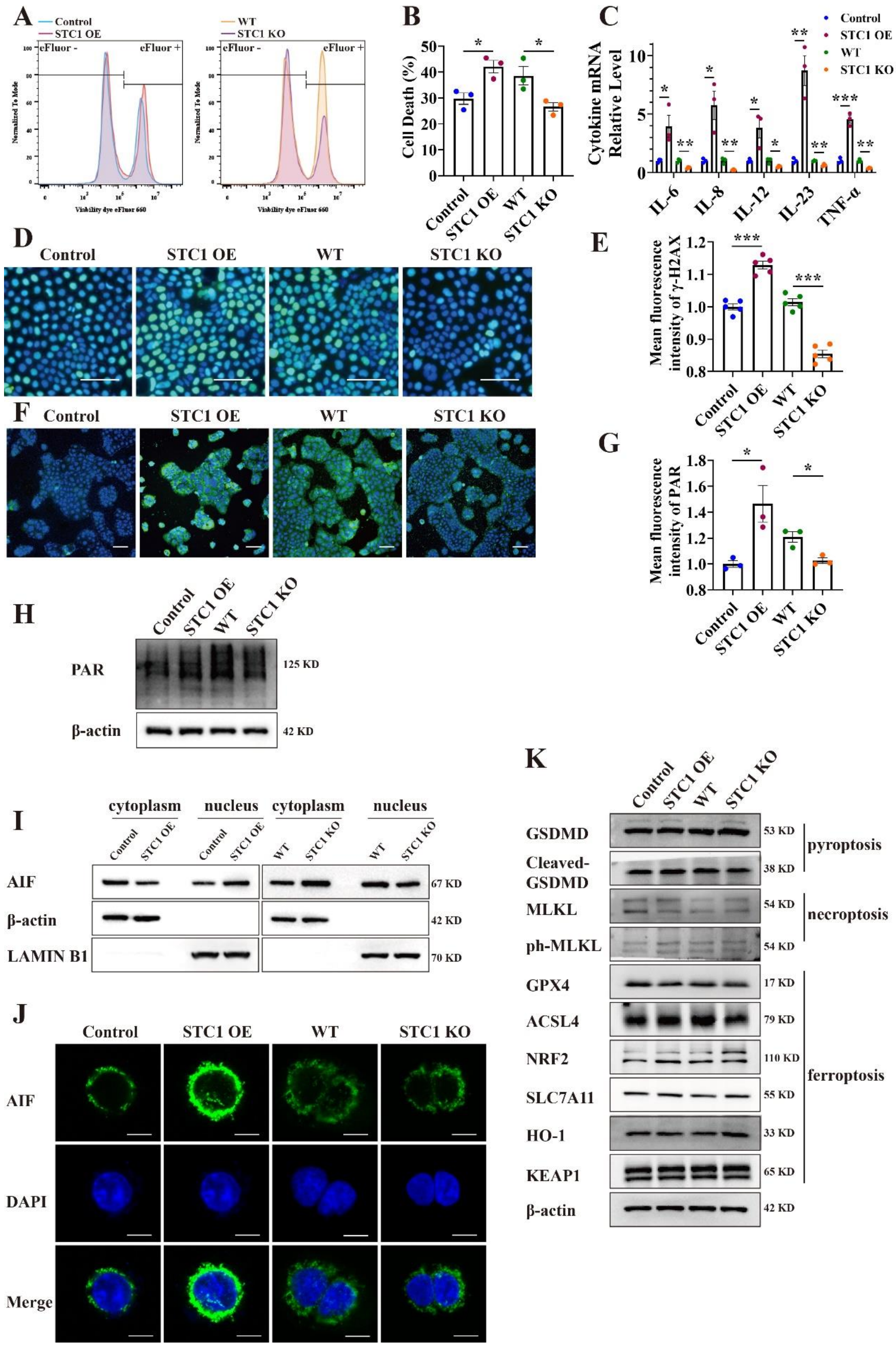

28

29 **Figure S3. STC1 intensifies parthanatos and aggravates oxidative stress-induced**

30 **inflammation in vitro.**

(A, B) Dye eFluor 660 marked dead cells after H_2O_2-stimulation in Control, STC1 OE, WT and STC1 KO Caco2 cells and was detected by flow cytometry. (C) The expression level of pro-inflammatory cytokine mRNA in H_2O_2-treated Caco2 cells was detected via qPCR. (D, E) Representative images and quantification of γ-H2AX-stained H_2O_2-treated Caco2 cells. Scale bars: 100μm. (F–H) The expression level of PAR in H_2O_2-treated Caco2 cells was detected via IF (F, G) and western blot (H). Scale bars: 100μm. (I, J) The nuclear translocation of AIF was detected by western blot (I) and IF (J). Scale bars: 10μm. (K) The expression level of cell death markers was detected in H_2O_2-treated STC1 OE and STC1 KO NCM460 cells. Data were expressed as mean ± SEM. * $p<0.05$, ** $p<0.01$, *** $p<0.001$.

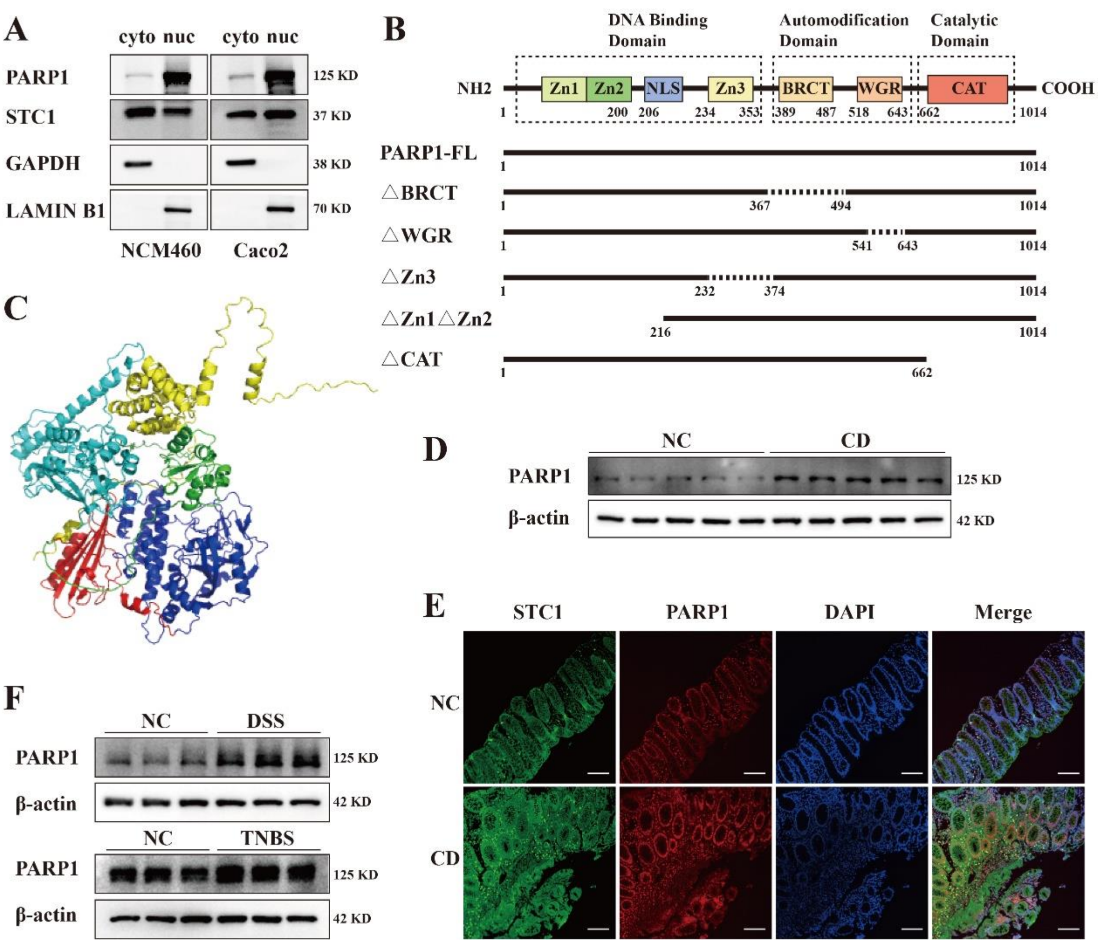

Figure S4. The interaction between PARP1 and STC1 protein.

(A) The subcellular distribution of STC1 and PARP1 protein in NCM460 and Caco2 cell lines was detected via western blot. (B) The schematic diagram of full-length PARP1 protein and truncated PARP1 protein. (C) The interaction between PARP1 and STC1 protein. PARP1 DNA binding domain (cyan), BRCT domain (green), WGR domain (red), CAT domain (blue). STC1 protein (yellow). (D) PARP1 protein expression in human colonic mucosa was detected via western blot. (E) Representative IF images of STC1- and PARP1-stained NC and CD colon mucosa sections. Scale bars: 100μm. (F) PARP1 protein expression in DSS- and TNBS-induced colitis models was detected via western blot.

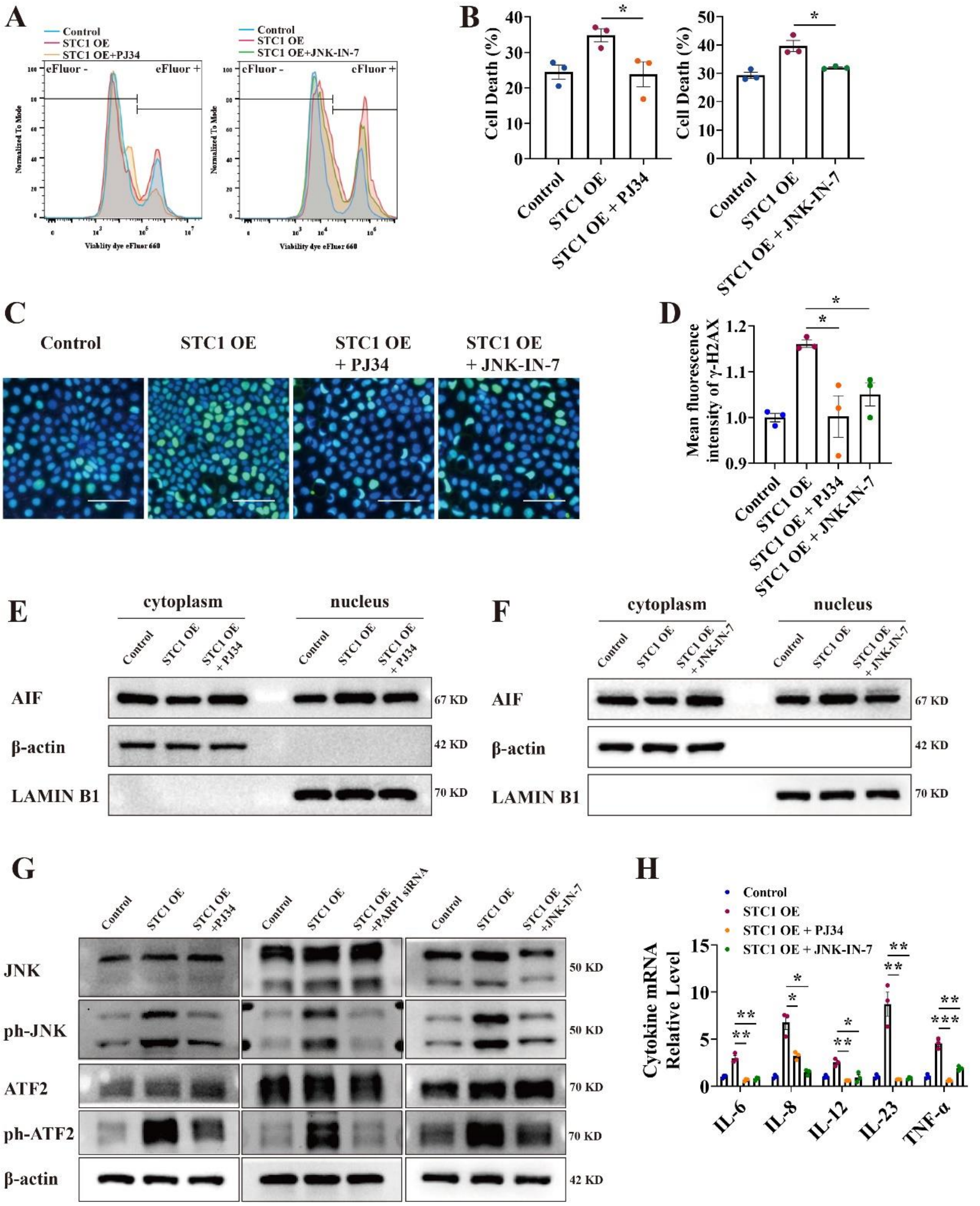

Figure S5. Inhibiting PARP1 and JNK attenuates parthanatos and oxidative stress-induced inflammation.

(A, B) After pretreating cells with PARP inhibitor PJ34 (5μM, 24 h) or JNK inhibitor JNK-IN-7 (3μM, 24 h), eFluor 660 was used in the identification of dead Caco2 cells in oxidative stress and detected by flow cytometry. (C, D) Representative IF images

and quantification of γ-H2AX-stained H_2O_2-treated Caco2 cells after PJ34 or JNK-IN-7 treatment. Scale bars: 100μm. (E, F) The nuclear translocation of AIF after PJ34 (E) or JNK-IN-7 (F) pretreatment was detected by western blot. (G) The expression level of JNK pathway protein after PJ34, PARP1 siRNA, and JNK-IN-7 pretreatment was detected by western blot. (H) The expression level of pro-inflammatory cytokine mRNA in H_2O_2-treated Caco2 cells after PJ34 or JNK-IN-7 pretreatment. Data were expressed as mean ± SEM. * p <0.05, ** p <0.01, *** p <0.001.

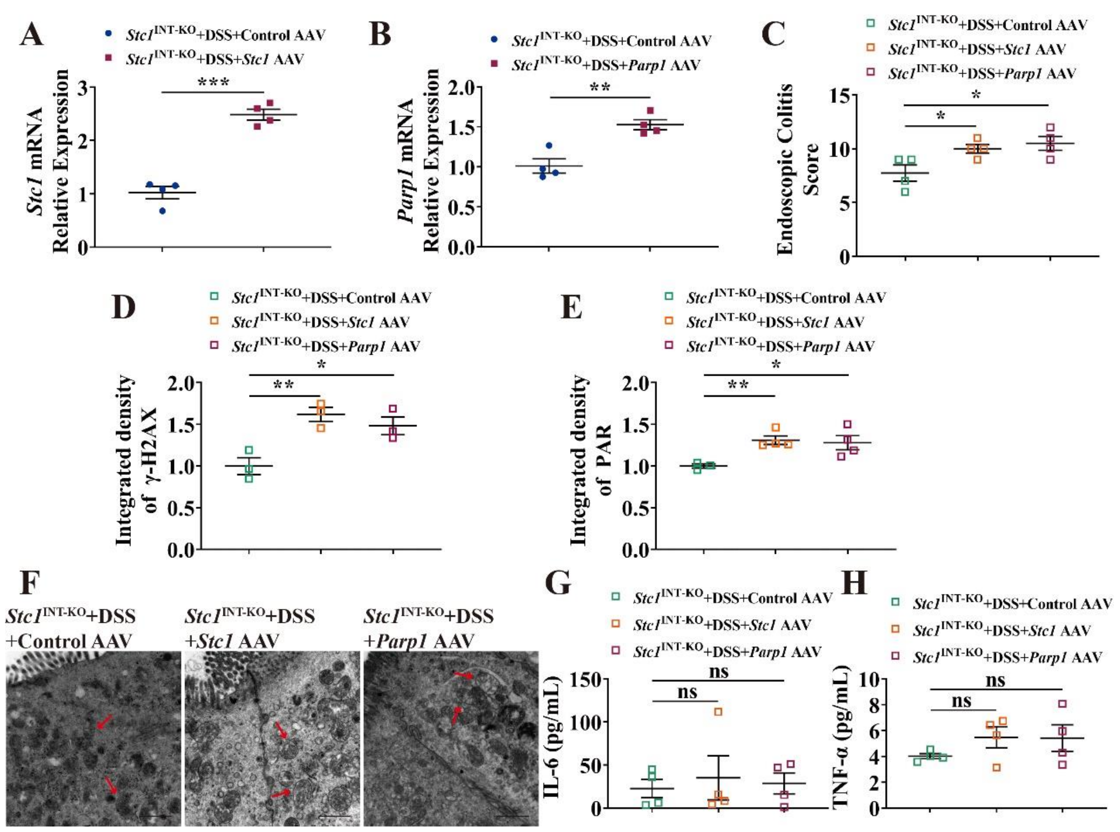

Figure S6. Restoring *Stc1* and *Parp1* in vivo aggravates DSS-induced mice colitis. (A, B) The expression level of *Stc1* and *Parp1* mRNA in the colonic tissues of *Stc1*$^{\text{INT-KO}}$ mice after intraperitoneal AAV injection. *Stc1*$^{\text{INT-KO}}$+DSS+Control AAV, n=4; *Stc1*$^{\text{INT-KO}}$+DSS+*Stc1* AAV, n=4; *Stc1*$^{\text{INT-KO}}$+DSS+*Parp1* AAV, n=4. (C) Endoscopic

colitis score of DSS-treated *Stc1*$^{\text{INT-KO}}$ mice after peritoneal injection of *Stc1*-overexpressing, *Parp1*-overexpressing or control AAV, was used to evaluate colitis severity observed under murine colonoscopy. *Stc1*$^{\text{INT-KO}}$+DSS+Control AAV, n=4; *Stc1*$^{\text{INT-KO}}$+DSS+*Stc1* AAV, n=4; *Stc1*$^{\text{INT-KO}}$+DSS+*Parp1* AAV, n=4. (D) The quantified fluorescent density of γ-H2AX-stained mice colon sections. *Stc1*$^{\text{INT-KO}}$+DSS+Control AAV, n=3; *Stc1*$^{\text{INT-KO}}$+DSS+*Stc1* AAV, n=3; *Stc1*$^{\text{INT-KO}}$+DSS+*Parp1* AAV, n=3. (E) The quantified fluorescent density of PAR-stained mice colon sections. *Stc1*$^{\text{INT-KO}}$+DSS+Control AAV, n=3; *Stc1*$^{\text{INT-KO}}$+DSS+*Stc1* AAV, n=4; *Stc1*$^{\text{INT-KO}}$+DSS+*Parp1* AAV, n=4. (F) TEM detected the deformation of mitochondria (marked by red arrows) in mice colonic epithelial cells. Scale bars: 1μm. (G, H) The expression level of IL-6 (G) and TNF-α (H) protein in mice serum was detected by multiELISA. *Stc1*$^{\text{INT-KO}}$+DSS+Control AAV, n=4; *Stc1*$^{\text{INT-KO}}$+DSS+*Stc1* AAV, n=4; *Stc1*$^{\text{INT-KO}}$+DSS+*Parp1* AAV, n=4. Data were expressed as mean ± SEM. * $p<0.05$, ** $p<0.01$, *** $p<0.001$.

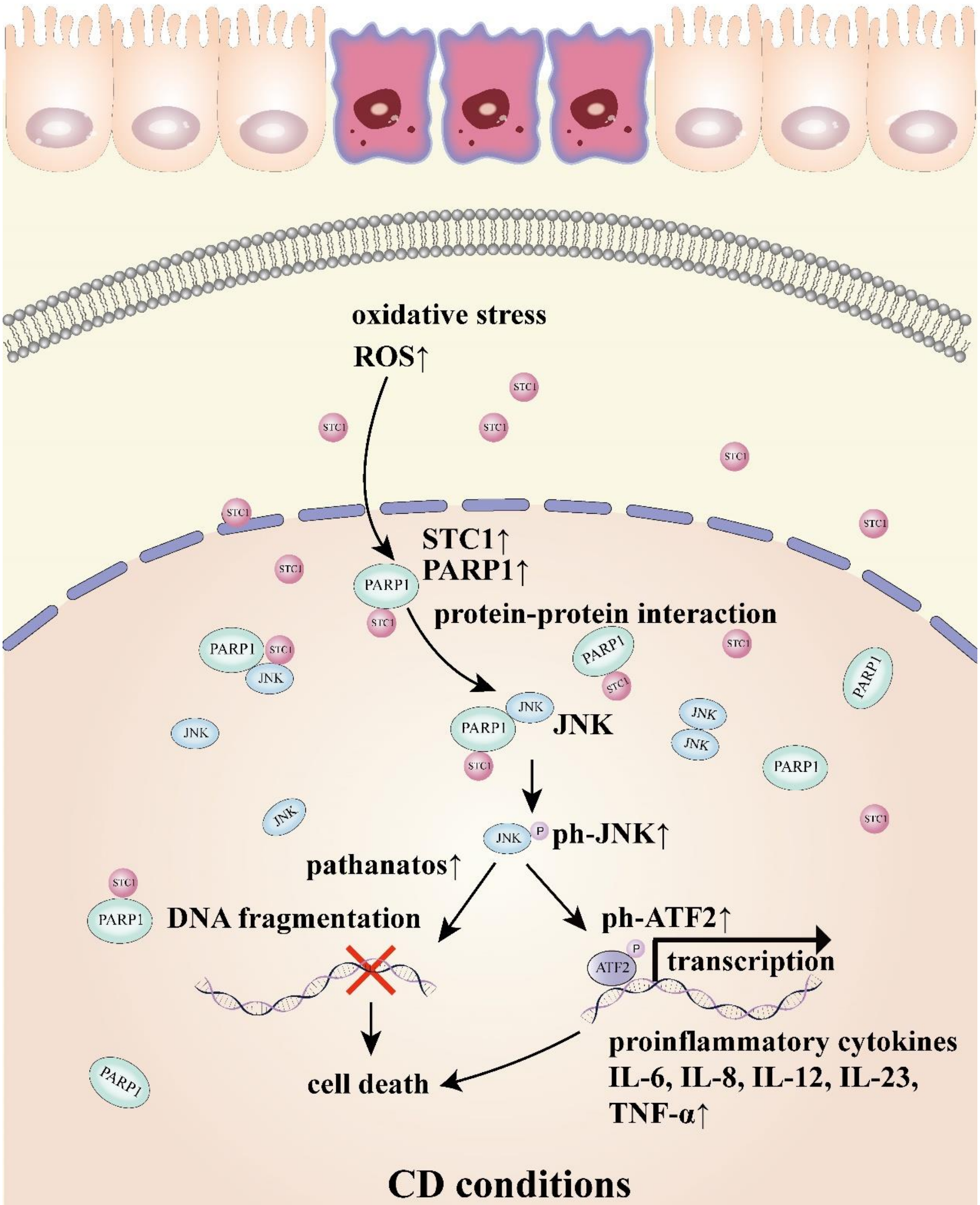

Figure S7. Schematic illustration of STC1-PARP1-JNK pathway.

Table S1. Antibodies

Antibody	Application	Cat.	Company
STC1	WB (1:1000), IHC (1:1000)	ab229477	abcam
STC1	IF (1:30), IP (1:25)	sc-293435	Santa Cruz
PARP1	WB (1:1000), IF (1:500)	ab191217	abcam
PARP1	IP (1:25)	sc-8007	Santa Cruz
β-actin	WB (1:1000)	4970	CST
LAMIN B1	WB (1:1000)	ab16048	abcam
γ-H2AX	IF (1:250)	ab81299	abcam
AIF	WB (1:1000), IF (1:500)	ab32516	abcam
PAR	WB (1:1000)	4335-MC-100	Bio-Techne
PAR	IF (1:100)	NBP2-89039	Novus
JNK	IP (1:25)	sc-137018	Santa Cruz
JNK	WB (1:1000)	9252	CST
Phospho-JNK	WB (1:1000)	4668	CST
ATF2	WB (1:1000)	35031	CST
Phospho-ATF2	WB (1:1000)	15411	CST
Anti-rabbit IgG (HRP-linked)	WB (1:2000)	7074	CST
Anti-mouse IgG (HRP-linked)	WB (1:2000)	7076	CST
Anti-rabbit IgG (H+L), F(ab')2 Fragment (Alexa Fluor® 488 Conjugate)	IF (1:500)	4412	CST
Donkey anti-Mouse IgG (H+L) Highly Cross-Adsorbed Secondary Antibody, Alexa Fluor™ 488	IF (1:500)	A21202	Invitrogen
Goat anti-Rabbit IgG (H+L) Cross-Adsorbed Secondary Antibody, Cyanine5	IF (1:500)	A10523	Invitrogen
SLC7A11/xCT	WB (1:1000)	A2413	Abclonal
ACSL4/FACL4	WB (1:1000)	ab155282	Abcam
GPX4	WB (1:1000)	ab125066	Abcam
HO-1	WB (1:1000)	ab68477	Abcam
Keap1	WB (1:1000)	ab227828	Abcam
Nrf2	WB (1:1000)	16396-1-AP	Proteintech
P-MLKL (S358)	WB (1:1000)	ab187091	Abcam
MLKL	WB (1:1000)	ab243142	Abcam
GSDMD	WB (1:1000)	ab210070	Abcam
cleaved GSDMD (N-terminal)	WB (1:1000)	ab215203	Abcam
His tag	WB (1:2000)	AF5060	Beyotime
DYKDDDDK tag	WB (1:2000)	80010-1-RR	Proteintech
HA tag	WB (1:2000)	51064-2-AP	Proteintech

Table S2. Primers

Gene	Forward Primer (5'-3')	Reverse Primer (5'-3')
Human STC1	GAAGTGGTTCGTTGCCTCAA	CGAATGCTTTTCCCTGAGTGT
Human PARP1	GGCAAGCACAGTGTCAAAGG	GAAATCCCGGTCCCAAGAGG
Human β-actin	CTAAGTCATAGTCCGCCTAGAAGCA	TGGCACCCAGCACAATGAA
Human IL-6	TTCGGTCCAGTTGCCTTCTC	CTGAGATGCCGTCGAGGATG
Human IL-8	ACTCCAAACCTTTCCACCCC	TTCTCAGCCCTCTTCAAAAACTTC
Human IL-12α	GCACAGTGGAGGCCTGTTTA	GCCAGGCAACTCCCATTAGT
Human IL-23α	CCCAAGGACTCAGGGACAAC	AGAGAAGGCTCCCCTGTGAA
Human TNF-α	GCTGCACTTTGGAGTGATCG	TCACTCGGGGTTCGAGAAGA
Mouse STC1	ACACAGATGGGATGTACGACA	GGAAAGTCGAACACCTCCGA
Mouse PARP1	GCGGAGAAGACATTGGGTGA	ACCATCTTCTTGGACAGGCG
Mouse β-actin	CCACTGTCGAGTCGCGT	CCACGATGGAGGGGAATACAG
Mouse IL-6	GTCCTTCCTACCCCAATTTCCA	TAACGCACTAGGTTTGCCGA
Mouse IL-8	TGTTCACAGGTGACTGCTCC	AGCCCATAGTGGAGTGGGAT
Mouse IL-12α	TCTTCTCACCGTGCACATCC	TGGCCAAACTGAGGTGGTTT
Mouse IL-23α	AATGCTATGGCTGTTGCCCT	CACTGGATACGGGGCACATT
Mouse TNF-α	GTAGCCCACGTCGTAGCAAA	ACAAGGTACAACCCATCGGC

Table S3. Clinical characteristics of CD patients and healthy controls analyzed for STC1 mRNA expression.

	**CD patients** (n=41)	**Healthy controls** (n=25)
**Sex**		
Male	26 (63.4)	16 (64.0)
Female	15 (36.6)	9 (36.0)
**Age (years)**	28 (18-37)	30 (18-41)
**Disease location**		
L1 (ileal disease)	9 (22.0)	-
L2 (colonic disease)	8 (19.5)	-
L3 (ileocolonic disease)	24 (58.5)	-
**Sampling location**		
Ascending colon	8 (19.5)	4 (16.0)
Transverse colon	19 (46.3)	12 (48.0)
Descending colon	10 (24.4)	6 (24.0)
Sigmoid colon	4 (9.8)	3 (12.0)
**Disease behaviour**		
B1 (non-stricturing, non-penetrating)	28 (68.3)	-
B2 (stricturing)	6 (14.6)	-
B3 (penetrating)	7 (17.1)	-
**Perianal disease**	11 (26.8)	-
**Current therapy**		
5-Aminosalicyclic acid	8 (19.5)	-
Thiopurine	10 (24.4)	-
Corticosteroids	2 (4.9)	-
Biologic therapy	8 (19.5)	-
None	13 (31.7)	-

Continuous variables and categorical variables are described as median and n (%).

Table S4. Clinical characteristics of UC patients and healthy controls analyzed for STC1 mRNA expression

	UC patients (n=19)	Healthy controls (n=26)
**Sex**		
Male	11 (60.6)	10 (38.5)
Female	8 (39.4)	16 (61.5)
**Age (years)**	38.4 (17-62)	41.0 (23-56)
**Disease location**		
E1 (rectum)	0 (0.0)	-
E2 (left hemi-colon)	4 (21.1)	-
E3 (extensive colon)	15 (78.9)	-
**Sampling location**		
Ascending colon	0 (0.0)	1 (3.8)
Transverse colon	3 (15.8)	3 (11.5)
Descending colon	3 (15.8)	8 (30.8)
Sigmoid colon	6 (31.6)	7 (26.9)
rectum	7 (36.8)	7 (26.9)
**Mayo Endoscopic score**		
0	0 (0.0)	-
1	1 (5.3)	-
2	7 (36.8)	-
3	11 (57.9)	-
**Current therapy**		
5-Aminosalicyclic acid	10 (21.2)	-
Thiopurine	2 (10.5)	-
Corticosteroids	2 (10.5)	-
Biologic therapy	5 (24.2)	-
None	0 (27.2)	-

Continuous variables and categorical variables are described as median and n (%).

Table S5. Clinical characteristics of CD patients and healthy controls analyzed for IHC analysis.

	CD patients (n=33)	Healthy controls (n=7)
**Sex**		
Male	20 (60.6)	4 (57.1)
Female	13 (39.4)	3 (42.9)
**Age (years)**	29 (19-40)	30 (20-42)
**Disease location**		
L1 (ileal disease)	7 (21.2)	-
L2 (colonic disease)	7 (21.2)	-
L3 (ileocolonic disease)	19 (57.6)	-
**Sampling location**		
Ascending colon	9 (27.3)	2 (28.6)
Transverse colon	17 (51.5)	4 (57.1)
Descending colon	5 (15.2)	1 (14.3)
Sigmoid colon	2 (6.1)	0 (0.0)
**Disease behaviour**		
B1 (non-stricturing, non-penetrating)	20 (60.6)	-
B2 (stricturing)	6 (18.2)	-
B3 (penetrating)	7 (21.2)	-
**Perianal disease**	8 (24.2)	-
**Current therapy**		
5-Aminosalicyclic acid	7 (21.2)	-
Thiopurine	8 (24.2)	-
Corticosteroids	1 (3.3)	-
Biologic therapy	8 (24.2)	-
None	9 (27.2)	-

Continuous variables and categorical variables are described as median and n (%).
